# Antimicrobial Contribution of Chitosan Surface-Modified Nanoliposomes Combined with Colistin against Sensitive and Colistin-Resistant Clinical *Pseudomonas aeruginosa*

**DOI:** 10.3390/pharmaceutics13010041

**Published:** 2020-12-30

**Authors:** Valentina Laverde-Rojas, Yamil Liscano, Sandra Patricia Rivera-Sánchez, Ivan Darío Ocampo-Ibáñez, Yeiston Betancourt, Maria José Alhajj, Cristhian J. Yarce, Constain H. Salamanca, Jose Oñate-Garzón

**Affiliations:** 1 Facultad de Salud, Programa de Medicina, Universidad Santiago de Cali, Calle 5 No. 62-00, Cali 760035, Colombia; valentina.laverde00@usc.edu.co; 2 Facultad de Ciencias Básicas, Programa de Microbiología, Universidad Santiago de Cali, Calle 5 No. 62-00, Cali 760035, Colombia; yamil.liscano00@usc.edu.co (Y.L.); sandra.rivera04@usc.edu.co (S.P.R.-S.); ivan.ocampo00@usc.edu.co (I.D.O.-I.); yeiston.betancourt00@usc.edu.co (Y.B.); 3 Laboratorio de Salud Pública Departamental, Secretaria Departamental de Salud del Valle del Cauca, Gobernación del Valle del Cauca, Cali 760045, Colombia; 4 Laboratorio de Diseño y Formulación de Productos Químicos y Derivados, Departamento de Ciencias Farmacéuticas, Facultad de Ciencias Naturales, Universidad Icesi, Cali 760035, Colombia; mariajoalhajj@hotmail.com (M.J.A.); cjyarce@icesi.edu.co (C.J.Y.)

**Keywords:** colistin, nanoliposomes, MDR-Bacteria, chitosan

## Abstract

Colistin is a re-emergent antibiotic peptide used as a last resort in clinical practice to overcome multi-drug resistant (MDR) Gram-negative bacterial infections. Unfortunately, the dissemination of colistin-resistant strains has increased in recent years and is considered a public health problem worldwide. Strategies to reduce resistance to antibiotics such as nanotechnology have been applied successfully. In this work, colistin was characterized physicochemically by surface tension measurements. Subsequently, nanoliposomes coated with highly deacetylated chitosan were prepared with and without colistin. The nanoliposomes were characterized using dynamic light scattering and zeta potential measurements. Both physicochemical parameters fluctuated relatively to the addition of colistin and/or polymer. The antimicrobial activity of formulations increased by four-fold against clinical isolates of susceptible *Pseudomona aeruginosa* but did not have antimicrobial activity against multidrug-resistant (MDR) bacteria. Interestingly, the free coated nanoliposomes exhibited the same antibacterial activity in both sensitive and MDR strains. Finally, the interaction of colistin with phospholipids was characterized using molecular dynamics (MD) simulations and determined that colistin is weakly associated with micelles constituted by zwitterionic phospholipids.

## 1. Introduction

Colistin (CST) is an antimicrobial peptide that was reintroduced into clinical practice as a last resort for the treatment of infections caused by multi-drug resistant (MDR) Gram-negative bacteria [1]. CST is a cyclic heptapeptide consisting of a tripeptide side-chain acylated at the N terminus by a fatty acid tail and has a cationic charge at neutral pH [2]. This antibiotic has been used to control and prevent infectious diseases in animals for decades, but its excessive use has contributed to the emergence of CST-resistant Enterobacteriaceae [3,4], and possibly to the horizontal transmission of CST resistance from farm animals to humans [5]. The plasmid-mediated resistance to CST via carriage *mcr-1* was first described in 2016 [6], and has been propagated throughout several countries, becoming a serious public health problem, especially with the coexistence of other resistance genes such as Extended spectrum beta-lactamases (ESBL) and New Delhi metallo-beta-lactamase (NDM) [7]. Therefore, this situation poses a major challenge for the treatment of life-threatening Gram-negative bacterial infections.

*Pseudomona aeruginosa* is an opportunistic pathogen that causes life-threatening infections, such as sepsis and pneumonia [8]. It is frequently found in hospital settings and uses several mechanisms of natural resistance to multiple antibiotics. In this respect, infections caused by MDR clinical isolates of *P. aeruginosa* have increased patient mortality and morbidity in intensive care units [8]. Therefore, MDR strains of *P. aureginosa* resistant to CST is a serious risk to human health that must be urgently addressed. In fact, *P. aeruginosa* is categorized as priority 1 in the list of bacteria for which new antibiotics are urgently needed, according to the World Health Organization (WHO) [9].

The disproportionate relationship between the emergence and dissemination of resistance mechanisms and the development of new molecules with antimicrobial properties is of great concern to public health. Therefore, it is important to consider innovative ways to face this resistance problem, such as the application of nanotechnology, which is an interesting alternative to usual antibiotic development approaches [10,11,12,13]. In an in vitro study, the resistance of methicillin-resistant *Staphylococcus aureus* (MRSA) strains to ampicillin loaded inside Eudragit^®^ polymer-coated nanoliposomes was markedly decreased [14], suggesting that the antimicrobial properties of the Eudragit^®^ polymer contributed synergistically to the effect of the ampicillin. On the other hand, Chitosan is a biocompatible, biodegradable and non-toxic linear cationic biopolymer obtained from the deacetylation of chitin under alkaline conditions [15]. Chitosan is a polymer of interest for the development of nanoparticle-based drug delivery, which was approved as generally recognized as safe (GRAS) by the US Food and Drug Administration (US FDA) [16]. The degree of deacetylation and the molecular weight of chitosan affect its physicochemical properties and consequently its biological activity. For example, highly deacetylated chitosan is more biologically active than chitin and less deacetylated chitosan [17].

Molecular dynamics simulations provide valuable and reliable information about the interaction of peptides with membrane models [18,19], which is useful to understand the behavior of CST in relation to liposomal systems. An advantage of using micelles as opposed to lipid bilayers is the faster time scales of motion of dodecylphosphocholine (DPC) lipids [20]. The interaction of the peptide with the lipid macromolecular assembly induces a much faster response in micelles as opposed to bilayers and captures the essential features that modulate the peptide–membrane interaction [20]. 

Therefore, we evaluated the antimicrobial effect of the peptide CST in the presence of self-assembling nanosystems. The stages of the study were—(i) isolation of MDR clinical isolates of *P. aeruginosa* and their phenotypic characterization, (ii) characterization of CST in solution using in silico and experimental methods, (iii) development and characterization of liposomal nanosystems, and (iv) antimicrobial evaluation of the CST combined with highly deacetylated (>90%) chitosan-coated nanoliposomes against both susceptible and MDR clinical isolates of *P. aureginosa*.

## 2. Materials and Methods 

### 2.1. Certified Bacterial Strains and Chemicals

*P. aeruginosa* ATCC^®^ 27853 ™ was obtained from the American Type Culture Collection (ATCC; Rockville, MD, USA). Muller Hinton Cation-Adjusted Broth and Colistin Sulfate were purchased from Merck (Darmstadt, Germany). Cholesterol and dioleoyl phosphatidyl ethanolamine (DOPE) were obtained from Avanti Polar Lipids (Alabaster, AL, USA). Soy lecithin (Medick) was purchased from a local pharmacy (Cali-Colombia) and characterized in a recent study [21]. Chitosan with a deacetylation degree of >90% (MW = 477 KDa) was provided by the Laboratory of Design and Formulation of Chemicals and Derivatives of the Icesi University [22].

### 2.2. Isolation of Resistant P. aeruginosa and Phenotypic Characterization

Three MDR clinical isolates of *P. aureginosa* encoded as Pa*01MDR*, Pa*02MDR*, and Pa*03MDR*, and one antibiotic susceptible *P. aureginosa* clinical isolate (Pa*wt*) were evaluated in this study. The antibiotic susceptibility, phenotypic characterization of the resistance of clinical isolates, and the minimal inhibitory concentration (MIC) for each antibiotic were confirmed using the VITEK^®^ 2 Antimicrobial Susceptibility Testing card (VITEK^®^ N272 -AST) in the VITEK^®^ system 2 (Ref. 414164, Biomerieux), according to the clinical cut-off points defined by the Clinical Laboratory Standards Institute (CLSI) [23]. The antimicrobial agents used in the phenotypical characterization included piperacillin/tazobactam (TZP), ceftazidime (CAZ), Cefepim (FEP), Doripenem (DOR), Imipenem (IPM), Meropenem (MEM), Amikacin (AMK), Gentamicin (GEN), Ciprofloxacin (CIP), and Colistin (CST), were evaluated. The resistance to CST was confirmed by performing a broth macrodilution test in glass tubes and the in vitro MIC was determined according to the clinical breakpoints defined by the CLSI [24]. The *P. aeruginosa* ATCC^®^ 27853 ™ strain was used as a reference strain.

### 2.3. Surface Tension Measurements

Measurement of CST surface tension was carried out using a surface tension meter (OCA15EC Dataphysics Instruments, Filderstadt, Germany) with the appropriate driver software (version 4.5.14 SCA22). The capture of the suspended droplet was recorded using an IDS video camera [25] and the solutions were prepared using dilutions from 5 mg/mL of reconstituted colistin with ultrapure water and phosphate-buffered saline at pH 7.2 (PBS, 138 mM NaCl, 3 mM KCl, 1.5 mM NaH_2_PO_4_, 8.1 mM Na_2_HPO_4_).

### 2.4. Chitosan-Coated Liposome Combined with CST

#### 2.4.1. Preparation of the Chitosan-Coated Liposomes with CST

The liposomes were constructed using the ethanol injection method [25]. Briefly, the organic phase (ethanolic solutions of washed soy lecithin, cholesterol, and DOPE with a ratio of 3:3:1, respectively) was added to the aqueous phase (CST, 100 µg/mL in PBS). Equal volumes were used for the aqueous and organic phases. The resulting mixture was vortexed for one minute to form the liposomes. Then, the liposomal suspension was diluted in ultrapure water at a proportion of 1:4. Later, 1 mL of an aqueous solution of chitosan (1 × 10^−4^ M, pH = 7.0) with a degree of deacetylation >90% was added to 1 mL of the liposomal dispersion free and combined with CST at a rate of 50 µL/min. Finally, the mixture was left under constant magnetic stirring at 300 rpm for 8 h in a closed polypropylene beaker.

#### 2.4.2. Physicochemical Characterization of Liposomal Systems

The particle size and zeta potential of the liposomes were determined using a Zetasizer Nano ZSP (Malvern Instrument, Cestershire, UK) with an He/Ne red laser (633 nm). The particle size was measured by dynamic light scattering (DLS) using a quartz flow cell (ZEN0023) and applying a light scattering angle from 173° to 25°. On the other hand, the zeta potential was measured using a disposable folded capillary cell (DTS1070). The instrument reports the particle size as the mean particle diameter (z-average), and the polydispersity index (PDI) ranges from 0 (monodisperse) to 1 (very wide distribution). All measurements were performed in triplicate after dilution of the freshly prepared liposome suspension in ultrapure water at a ratio of 5: 5000 *v/v*.

### 2.5. Molecular Dynamics of Colistin and Nanoliposomes 

#### 2.5.1. Construction of the Colistin 3D Structure

The SDF 2D structure of CST (Polymyxin E) was obtained from the Pubchem database (https://pubchem.ncbi.nlm.nih.gov/compound/5311054). The 3D structure of CST was constructed using Avogadro version 1.2 (https://avogadro.cc/) optimized with General Amber Force Field (GAFF) and steepest descent algorithm to obtain a PDB file for molecular dynamics (MD) [26,27].

#### 2.5.2. CST in Aqueous Solution and Construction of CST inside of DPC Micelles

The CHARMM-GUI platform [28] was used to develop a micelle hydrated system consisting of 100 units of phospholipid DPC surrounding CST. DPC monolayers were constructed to visualize and understand the stability of one molecule of CST inside the micelle. GROMACS software version 2019.3 (University of Groningen, Groningen, The Netherlands) was used for molecular dynamics simulation [29]. The ion Monte Carlo method was used with 0.15 M NaCl, with a water thickness of 22.5 Å and CHARMM36m as a forcefield, which is suitable for describing the distribution of molecules within large systems such as micelles [30]. CST parametrization was performed with a CHARMM-GUI PDB reader [31] using par_all36_prot_lipid.prm. The behavior of CST in aqueous solution and inside of the micelle was compared.

#### 2.5.3. Minimization Energy, Equilibration and MD

To ensure the absence of steric clashes between the CST and the micelle, the system was adjusted by heating to a temperature of 310 K at 1 fs (femtosecond)/step for 75 ps (picoseconds), relaxing the structure by energy minimization for 300 ps at a rate of 2 fs/step. The energy minimization of the system was obtained with the steepest descent algorithm (tolerance value of 1000 kJ⋅mol^−1^ nm^−1^) in 5000 steps. The Berendsen algorithm was used to equilibrate the temperature and pressure of the system. After the system equilibration, the MD was run for data collection during 10 ns, using the Nose–Hoover and Parrinello–Rahman algorithms to adjust the temperature and pressure. The particle mesh Ewald summation was applied to handle the long-range electrostatic interactions [32]. 

#### 2.5.4. MD and Interaction Analysis

The software VMD (Visual Molecular Dynamics, University of Illinois, Chicago, IL, USA) was used to visualize the simulation [33]. In order to determine the root mean square deviation (RMSD) and the hydrogen bond formation between colistin and the model system, a simulation analysis was performed using gromacs. Finally, the interactions were manually analyzed using Discovery Studio Visualizer version Client 2020 (https://discover.3ds.com/discovery-studio-visualizer-download) and Ligplot+ [34].

### 2.6. Antimicrobial Activity

The antimicrobial susceptibility was assessed through a broth macrodilution test in glass tubes following the suggestions proposed by the CLSI [23]. The clinical isolates of *P. aeruginosa* previously characterized as susceptible and resistant to CST were incubated at 37 °C for 18–24 h on Brain Heart Infusion (BHI) agar. One colony was initially resuspended in sterile water to reach a turbidity of 0.5 McFarland (approximately 1–4 × 10^8^ colony forming units (CFU)/mL). The bacterial suspension was then diluted with Mueller–Hinton broth adjusted with cations to reach a concentration of approximately 2–7 × 10^5^ CFU/mL. Subsequently, 1 mL of bacterial suspension and 1 mL of free CST were mixed in a glass tube using serial concentrations from 256 to 0.25 µg/mL and incubated at 37 °C for 18 h. For CST combined with nanoliposomes, the same volumes and the same incubation conditions were used, but the initial concentration of CST was adjusted to 64 µg/mL to be diluted in half to 0.25 µg/mL. PBS was used as a negative control and the ATCC strain was used as a reference to ensure reproducibility of the assays. After incubation, the MIC was determined by visual analysis. Assays were performed in triplicate.

## 3. Results and Discussion

### 3.1. Phenotypic Characterization of Clinical Isolates of MDR P. aeruginosa

The antibiotic susceptibility and resistance profiles of *P. aeruginosa* clinical isolates were constructed using six conventional antimicrobial categories (Table 1). The Pa*01MDR* strain was resistant to penicillin + β-lactamase inhibitor (TZP), to extended-spectrum cephalosporins (CAZ, FEP), to carbapenems (DOR, IPM, and MEM), and to polymyxins (CST), but it was susceptible to aminoglycosides (AMK, GEN) and fluoroquinolones (CIP). Pa*02MDR* and Pa*03MDR* clinical isolates were resistant to all the antibiotics evaluated according to the clinical breakpoints defined by the CLSI guidelines [23].

### 3.2. Surface Tension Measurements

**Figure 1:**
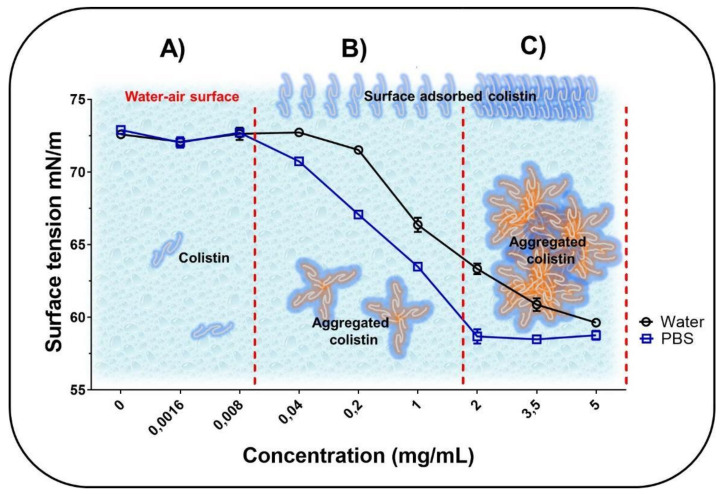
Characterization of the surface tension of Colistin (CST) in aqueous medium at different concentrations ranging from 0 to 0.008 mg/mL (**A**), from 0.008 to 2 mg/mL (**B**) and from 2 to 5 mg/mL (**C**).

The surface tension at different CST concentrations is shown in Figure 1.

The behavior of the surface tension against different concentrations of the CST follows a profile typical of amphiphilic molecules, which is consistent with the structural nature of CST. Besides, at low CST concentrations (<0.008 mg/mL in PBS and <0.04 mg/mL in water), the surface tension tends to remain constant at a typical value of ~73 × 10^−3^ mN/m at 25 °C (Figure 1A). This suggests that at these concentrations, the CST is mainly solubilized within the solution bulk with very few chains in the surface area. However, increasing the peptide concentration from 0.04 to 5 mg/mL in water and from 0.008 to 2 mg/mL in PBS leads to a decrease in surface tension from ~73 to ~60 mN/m and from ~73 to ~59 mN/m, respectively (Figure 1B). Therefore, at such concentrations, CST may begin to localize at the air–water surface, as well as in the solution bulk, where other association effects between the different peptide chains also start to take place, forming CST–CST aggregates. In this form, the peptide acts as a surfactant in an aqueous medium, decreasing the cohesive forces between water molecules located at the interface [35]. Finally, the CST–CST aggregates prevailed at the higher peptide concentrations in water (5 mg/mL) and PBS (2 mg/mL) (Figure 1C). Notably, the CST–CST aggregates were formed at a lower concentration in PBS than in ultrapure water. This may be due to the charge shielding effect generated when the phosphate anions of PBS are attracted to the cationic groups of CST until the peptide is electrically neutralized. Thus, the low polarization of the peptide interface promotes rapid and easy aggregation of CST–CST [36]. Therefore, depending on whether the CST is free or added, it is possible to obtain a greater or lesser antimicrobial effect. However, this mechanism is not fully elucidated, even though the few studies focused on evaluating this type of effect found that free forms have more antimicrobial activity than added forms [37,38].

### 3.3. Physicochemical Characterization of Liposomal Systems

**Figure 2:**
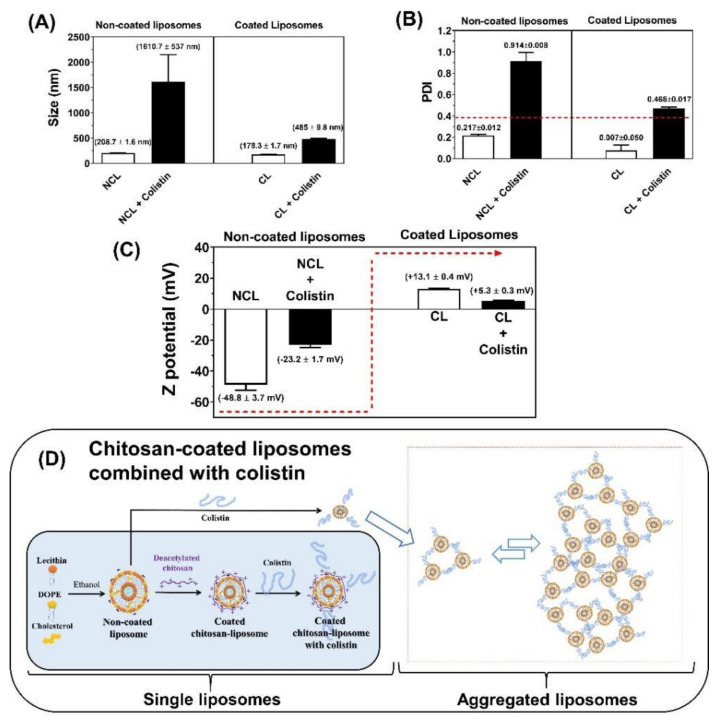
Physicochemical characterization of uncoated and chitosan-coated liposomes in the presence and absence of CST. (**A**) Particle size, (**B**) Polydispersity index-PDI, (**C**) Zeta potential, (**D**) Schematic representation of the liposomal coating process and its interaction with CST. NCL—Non-coated liposomes; CL—coated liposomes.

The characteristics of the uncoated and chitosan-coated liposomal systems in the presence and absence of CST are shown in Figure 2. For the uncoated liposomes, the particle size was 208.7 ± 1.6 nm, which increased considerably to 1610.7 ± 537.0 nm when combined with CST. On the contrary, the free chitosan-coated liposomes had a particle size of 178.3 ± 1.6 nm, while combined with CST they reached 485.0 ± 9.8 nm, showing a compaction effect compared to uncoated liposomes (Figure 2A). Likewise, the PDI of these systems strongly depends on the coating with chitosan and its combination with CST (Figure 2B). The PDI for the uncoated liposomes was 0.217 ± 0.012, but an abrupt increase was observed when they were combined with CST (Figure 2B). In the case of chitosan-coated liposomes, the combination with CST also caused an increase of the PDI from 0.007 ± 0.005 to 0.468 ± 0.017 (Figure 2B). As shown in Figure 2C, the zeta potential was −48.8 ± 3.7 mV for the uncoated liposomes, and it increased to −23.2 ± 1.7 mV when they were combined with CST. This suggests that the positively charged CST locates on the liposomal surface, but the positive charge density is not sufficient to reverse the negative charge contributed by the phosphate groups of the phospholipids. Oppositely, the free chitosan-coated liposomes had a zeta potential of +13.1 ± 0.4 mV, which decreased slightly to +5.3 ± 0.3 mV when they were combined with CST.

All these results can be explained considering that under the methodology used, the liposomes based on lecithin, DOPE, and cholesterol are nanometric, very uniform in size, and with a negative surface potential, as previously described for such systems [14]. However, when these liposomes are coated with highly deacetylated chitosan, a slight size contraction, a greater homogenization of the size populations (to a point of high uniformity), and an inversion of the surface charge occur, indicating that the chitosan is completely deposited on the liposomal surface. These results suggest that the chitosan coating process occurs in a highly organized manner and forms a very well-defined system. Interestingly, there is not an additive effect between the positive charges of chitosan and CST on the zeta potential (Figure 2C), suggesting that there is a competition between both molecules for binding to the liposomal surface. Thus, when chitosan-coated liposomes are combined with CST, the latter would be displaced by chitosan forming a slightly more organized and less polydisperse system (Figure 2D). On the other hand, CST could mediate contacts between liposomes [39] or interacts differently with liposomes leading to increased size and subsequent changes in the aggregation indices of liposomes and to more polydisperse systems, which is consistent with the high dynamism of this peptide, which can form a balance between its chains and aggregates. 

### 3.4. Molecular Dynamics of CST Combined with Nanoliposomes

To complement the results obtained in the Section 3.3 and understand how CST interacts with uncoated nanoliposomes, micelle-type aggregation systems were built with DPC phospholipids (which is zwitterionic, like those that constitute nanoliposomal formulations) and the CST was initially placed inside the system. Although the nanosystems built above (Section 2.4.1) are lamellar liposomes, we simulated DPC micelles to elucidate the initial motion of the peptide upon insertion into the membrane because micelles are commonly used as bilayer membrane mimetics to study the interaction protein–membrane [40] and the data obtained from these two systems, bilayers and micelles, can be used in a complementary fashion [41].

**Figure 3:**
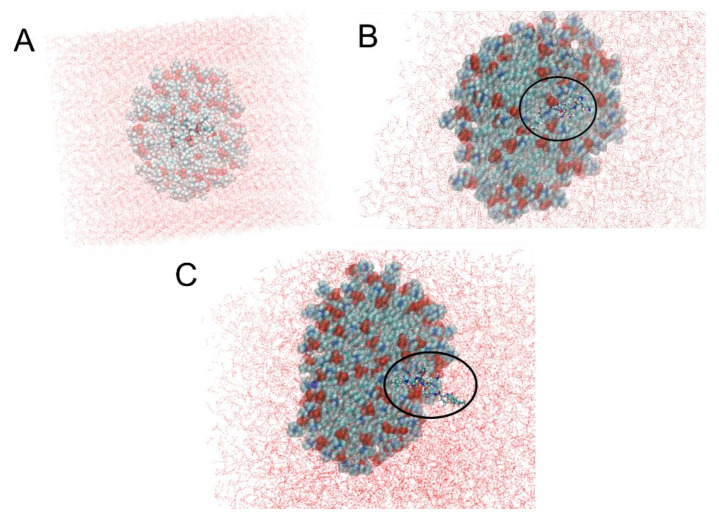
CST interaction with dodecylphosphocholine (DPC) micelle through 10 ns (**A**) DPC Micelle with CST at 0 ns. (**B**) DPC micelle with CST at 2 ns (**C**) DPC micelle with CST at 7 ns. Black circle highlights the CST.

After 7 ns, most of the CST comes out of the DPC micelle due to its strong hydrogen bond interaction with the water molecules surrounding the micelle. CST appears at 2 ns (Figure 3B) and is even more visible at 7 ns (Figure 3C). This is consistent with the results shown in Figure 2A,B since it reveals that CST is positioned on the external surface of nanoliposomes, thereby increasing the size and the Z potential value. Although the DPC headgroup carries a highly electronegative phosphate center comprised of four strong hydrogen-bond acceptor oxygen atoms [20], the binding between CST and DPC micelles is reduced.

**Figure 4:**
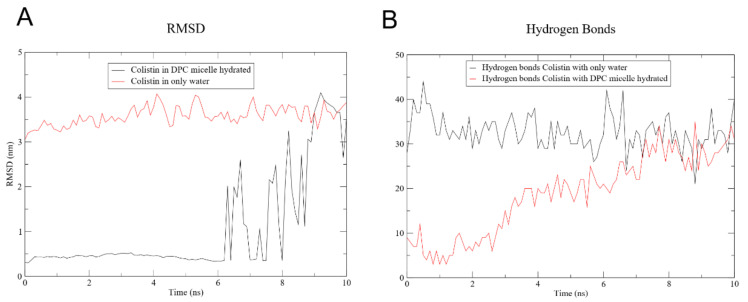
CST molecular dynamics (MD) analysis. (**A**) Root mean square deviation (RMSD) of the DPC micelle with CST and CST in water only. (**B**) Hydrogen bonds of DPC micelle with CST and CST in water only.

Figure 4A shows the RMSD between CST and the hydrated DPC micelle, and of CST immersed in water only. In the latter, the RMSD varies little, apparently because of the constant formation of hydrogen bonds between CST and water which remains at an average of 33 hydrogen bonds during the 10 ns (Figure 4B). Contrarily, the RMSD of the CST placed inside the micelle shows that it stays in the micelle for 7 ns, and the RMSD increases to approximately 3 nm, suggesting that CST migrates from the micelle core toward the water surrounding the DPC in agreement with what was shown in Figure 3. The number of hydrogen bonds with water molecules increases progressively until it reaches 20 at 7 ns. CST has six L-diaminobutyric acid residues, and five of these can form hydrogen bonds. Also, two threonine residues provide donor and acceptor atoms for the formation of relatively weaker hydrogen bonds. 

**Figure 5:**
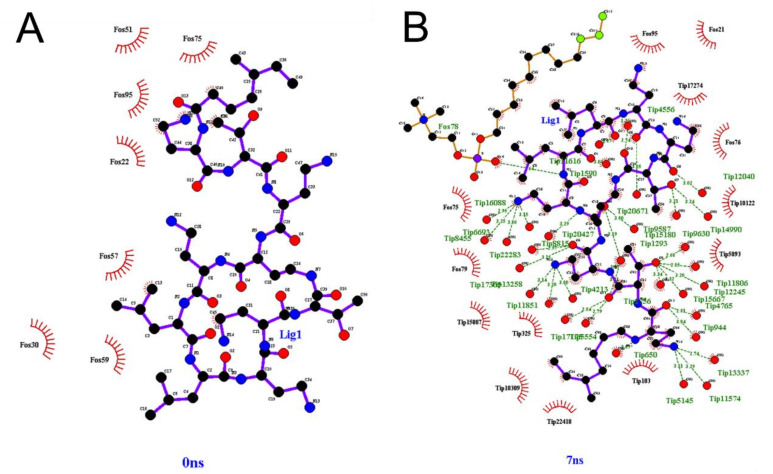
Interaction between CST and DPC micelle hydrated atoms (**A**) at 0 ns. (**B**) At 7ns. Fos: DPC molecule. Lig: CST. Tip: water molecule.

Interactions between CST and hydrated micelle by hydrogen bonds are shown in Figure 5. At 0 ns, no hydrogen bonds exist between CST and DPC (Figure 5A). The few hydrogen bonds formed are intramolecular for CST as seen in Supplement 1, where a minimal hydrophobic interaction with DPC is observed. The number of hydrogen bonds between CST and DPC remains almost constant, ranging between 7 and 10 interactions from 1 to 7 ns. Conversely, there are no interactions between CST and the water molecules surrounding the micelles at 0 ns, but they increase considerably as time passes (Figure 5B). There were also few hydrophobic interactions between CST and DPC, going from two interactions at 0 ns to one at 7 ns (Table S1), suggesting that the hydrophobic interactions between the acyl chain of CST and the hydrophobic region of the micelle is not enough to compensate the polar interactions between side chains and backbone polar groups of CST with water molecules.

### 3.5. Antimicrobial Activity

The antibacterial activities of free CST (CST), coated nanoliposome without CST (CL), and coated nanoliposome combined with CST (CL + CST) were evaluated against different susceptible and MDR *P. aeruginosa* clinical isolates (Table 2). CST exhibited a MIC of 2 µg/mL for both Pa ATCC 27853 and Pa*wt*. CST was initially attracted to the bacterial surface because of the electrostatic interaction between the positively charged αγ-diaminobutyric acid residues and the negatively charged phosphate groups of lipid A of lipopolysaccharide (LPS) [42]. There, CST displaces the divalent cations (Ca^2+^ and Mg^2+^) from the LPS layer, therefore increasing the fluidity and permeability of the membrane, leading to the release of cytoplasmic content and finally cell death [2]. On the other hand, MIC values for MDR clinical isolates ranged between 8 to 16 µg/mL. Resistance to CST is closely related to the loss of its affinity for LPS because of the neutralization of the negative charge of the phosphate groups of LPS by the addition of aminoarabinose and ethanolamine residues [43]. However, even though the surface membrane charge has been reduced, CST still exhibits antimicrobial activity against MDR strains at moderately higher concentrations (Table 2). This is because the membrane of CST-resistant bacteria is still negatively charged [44] due to anionic phospholipids that are exposed on the surface, which could interact electrostatically with cationic CST.

CL + CST reduced the MIC in susceptible strains by four-fold (Table 2). This antimicrobial contribution against *P. aeruginosa* is provided by highly deacetylated chitosan as reported in previous studies [22,45]. To explain these results, two hypotheses could be proposed—(i) cationic chitosan and CST may interact with the membrane simultaneously since both can be attracted by the anionic charge of the membrane [42,45,46]. This way, the destabilizing effect that chitosan adds to the effect of CST on the membrane could explain the decrease in MIC; (ii) while CST acts on the membrane, chitosan could penetrate the cell wall and membrane and travel to the nucleus where it could interfere with mRNA synthesis, causing a decrease in MIC [47].

However, CL + CST did not increase the antimicrobial effect of CST on MDR strains (Table 2). This can be explained according to the results obtained by the physicochemical characterization of the nanoliposomes and by the MD simulations (Figure 2 and Figure 3) where the localization of chitosan and colistin outside of the nanosystem was revealed. In this way, several cationic CST molecules remain in solution because they would not be attracted by the neutralized LPS of MDR strains [43], and thus, the cationic charge of CST would not be neutralized by the phosphate groups of LPS, unlike with susceptible strains. Therefore, in the CL + CST system, both cationic molecules CST and chitosan compete with each other as seen in Figure 2C, preventing the latter from encountering the bacteria because of electrostatic repulsion with CST and avoiding an additive antimicrobial effect, in contrast to that observed in sensitive strains. 

Interestingly, the nanoliposome coated with high degree deacetylated chitosan without CST (CL) showed antimicrobial activity in the third dilution equivalent to 1.25 × 10^−5^ M (5.96 µg/mL) (Table 2). A previous study reported an approximate MIC of chitosan with a degree of deacetylation of >90% against *P. aeruginosa* [45]. Our results suggests that chitosan has a similar antimicrobial effect in all strains regardless of the resistance level to CST and therefore, the decrease in the anionic charge magnitude of the bacterial surface by LPS modification in CST-resistant strains does not affect the antibacterial function of this polymer. In addition, the antibacterial effect of highly deacetylated chitosan is not altered by the presence of β-lactamases, carbapenemases, or by structural changes in prokaryotic ribosome and DNA gyrase. Studies have reported that chitosan exhibits a membranolytic effect against MDR bacteria [48,49], a consistent approach considering that the cell membrane remains anionic despite resistance to colistin [44], therefore, both sensitive and MDR strains could electrostatically attract chitosan to the surface. However, recent evidence revealed that chitosan penetrates the cell membrane of *P. aeruginosa*, releasing the cell contents and aggregating the residue cytoplasmic material into a mass. It was presumed that the cytoplasmic material was agglomerated by flocculation of chitosan after entering the cell [50], an effect that would overcome the resistance mechanisms mentioned above. Finally, so far, this finding could mean highly deacetylated chitosan is a potential antimicrobial agent to use against CST-resistant MDR strains.

## 4. Conclusions

CST behaves differently in water or PBS buffer, depending on its concentration. At low concentrations, it is solubilized in the bulk while at higher concentrations it behaves in two specific ways. First, it decreases the surface tension due to its adsorption at the air–aqueous medium interface. Second, it aggregates within the solution, establishing a condition of high dynamism between the aggregated and non-aggregated forms.

The liposomes have a homogeneous nanometric size and with negative zeta potentials, and when they are combined with the coating polymers (chitosan), they form more compact and homogeneous systems, but with positive zeta potential. Conversely, the combination of liposomal systems with CST leads to the formation of larger and more polydisperse systems, suggesting a random interaction between the liposomal surface (coated and uncoated) and the peptide. This result agrees with those obtained with in silico studies, which show that CST has a greater tropism towards the external aqueous medium than towards phospholipids. Based on this, we assume that CST amounts are in equilibrium between the internal and external environment of the nanoliposome. We will confirm this hypothesis in later studies using in silico lamellar systems. 

Finally, combining CST with coated nanoliposomes increased its antimicrobial activity by four-fold against sensitive *P. aeruginosa* but did not make any contribution against MDR strains. Interestingly, coated nanoliposomes without CST exhibited the same antimicrobial activity in susceptible and MDR strains. Thus, this polymer is considered a potential antibiotic that should be further explored against MDR *P. aeruginosa*. 

## Figures and Tables

**Table 1:** Bacterial resistance profile from phenotypic tests.

Scheme	MIC (μg/mL)									
	TZP	CAZ	FEP	DOR	IPM	MEM	AMK	GEN	CIP	CST
Pa ATCC	≤4/S	≤1/S	≤1/S	≤0.25/S	≤2/S	≤0.25/S	≤2/S	≤1/S	≤0.25/S	≤0.5/S
Pa*wt*	8/S	4/S	2/S	0.5/S	1/S	≤0.25/S	≤2/S	≤1/S	≤0.25/S	≤0.5/S
Pa*01MDR*	≥128/R	≥64/R	≥64/R	≥8/R	≥16/R	≥16/R	8/S	4/S	0.5/S	≥8/R
Pa*02MDR*	≥128/R	≥64/R	≥64/R	≥8/R	≥16/R	≥16/R	≥64/R	≥16/R	≥4/R	≥8/R
Pa*03MDR*	≥128/R	≥64/R	≥64/R	≥8/R	≥16/R	≥16/R	≥64/R	≥16/R	≥4/R	≥8/R

S = Susceptibility strain, R = Resistant strain.

**Table 2:** Antimicrobial activity (MIC) of CST, CST-free coated nanoliposomes (CL) and CST-coated nanoliposomes (CL + CST) against sensitive and multidrug resistant (MDR) strains of *P. aeruginosa.*

Strain	MIC (µg/mL)		
	CST	CL + CST	CL *
Pa ATCC 27853	2	0.5	5.96
Pa*wt*	2	0.5	5.96
Pa*01MDR*	8	8	5.96
Pa*02MDR*	16	16	5.96
Pa*03MDR*	8	8	5.96

* Final chitosan concentration coating the nanoliposomes.

## Data Availability

There are not limitations in the Data Availability Statement.

## Supplementary Materials

The following are available online at https://www.mdpi.com/1999-4923/13/1/41/s1, Table S1: Interactions between colistin and micelle hydrated system at 0 ns and 7 ns. LIG: colistin; H: hydrogen; O: oxygen; TIP: water; C: carbon.
