# Supplementary material for: Antimicrobial Contribution of Chitosan Surface-Modified Nanoliposomes Combined with Colistin against Sensitive and Colistin-Resistant Clinical Pseudomonas aeruginosa
Source: Pharmaceutics. 2020 Dec 30;13(1):41. doi: 10.3390/pharmaceutics13010041 (PMC7824406; doi:10.3390/pharmaceutics13010041)
Supplement: Supplementary file 1 [file pharmaceutics-13-00041-s001.pdf]

pharmaceutics

MDPI

Supplementary Material

Antimicrobial Contribution of Chitosan Surface-Modified Nanoliposomes Combined with Colistin against Sensitive and Colistin-Resistant Clinical *Pseudomonas aeruginosa*

Valentina Laverde-Rojas, Yamil Liscano, Sandra Patricia Rivera-Sánchez, Ivan Darío Ocampo-Ibáñez, Yeiston Betancurt, Maria José Alhajj, Cristhian J. Yarce, Constain H. Salamanca and Jose Oñate-Garzón

Supplement Table S1: Interactions between colistin and micelle hydrated system at 0 ns and 7 ns. LIG: colistin; H: hydrogen; O: oxygen; TIP: water; C: carbon

Molecule	Hydrogen bonds	Hydrophobic bond
Colistin 0ns	LIG1:H68 - :LIG1:O6; LIG1:H94 - :LIG1:O13; LIG1:H45 - :LIG1:O2; LIG1:H60 - :LIG1:O5; LIG1:H34 - :LIG1:O4; LIG1:H71 - :LIG1:O8	LIG1:C51 - FOS95; LIG1:C51 FOS95:C311
Colistin 7ns	LIG1:H37 - :TIP10122:OH2; LIG1:H58 - :TIP1293:OH2; LIG1:H64 - :TIP5893:OH2; LIG1:H68 - :TIP5893:OH2; LIG1:H74 - :TIP4765:OH2; LIG1:H75 - :TIP650:OH2; LIG1:H80 - :TIP8455:OH2; LIG1:H93 - :TIP13763:OH2; LIG1:H96 - :TIP9630:OH2; LIG1:O12 - TIP944:H2; LIG1:O2 - TIP11616:H2; LIG1:O8 - TIP12040:H1; LIG1:O4 - TIP1293:H1; LIG1:N16 - TIP13337:H1; LIG1:O13 - TIP13343:H1; LIG1:O4 - TIP15180:H1; LIG1:O1 TIP1590:H2; LIG1:O11 - TIP17175:H2; LIG1:O6 - TIP17365:H1; LIG1:O5 - TIP20671:H2; LIG1:O6 - TIP22283:H1; LIG1:O9 - TIP2756:H1; LIG1:O9 - TIP2756:H2; LIG1:O10 - TIP4556:H2; LIG1:O12 - TIP4765:H2; LIG1:O11 -TIP5554:H2; LIG1:O13 - TIP9059:H2; LIG1:O7 - TIP9587:H1; LIG1:H11 - :FOS78:O14; LIG1:H45 - :LIG1:N14; LIG1:H57 - :LIG1:O1; LIG1:H2 - :LIG1:O10; LIG1:H10 - :FOS78:O14; LIG1:H35 - :LIG1:O12; LIG1:H71 - :FOS76:O14; LIG1:O5 - FOS75:H11B; LIG1:O5 - FOS75:H12A; LIG1:O3 - FOS75:H13C; LIG1:O3 - FOS75:H15A; LIG1:O2 - FOS75:H15B; LIG1:O5 - FOS75:H15B	LIG1:C6 - FOS78
